# Financing repurposed drugs for rare diseases: a case study of Unravel Biosciences

**DOI:** 10.1186/s13023-023-02753-y

**Published:** 2023-09-12

**Authors:** Bechara Abouarab, Christian Bazarian, Zied Ben Chaouch, Andrew W. Lo, Guillermo Mourenza Gonzalez, Richard Novak, Frederic Vigneault

**Affiliations:** 1 Sloan School of Management, MIT, Cambridge, USA; 2 Department of Electrical Engineering and Computer Science, MIT, Cambridge, USA; 3 Computer Science and Artificial Intelligence Laboratory, MIT, Cambridge, USA; 4 Laboratory for Financial Engineering, MIT, Cambridge, USA; 5 Wyss Institute, Harvard University, Boston, USA; 6 Unravel Biosciences, Boston, USA; 7 Santa Fe Institute, Santa Fe, USA

**Keywords:** Early stage drug development, Drug repurposing, Rare disease therapeutics, Biotech venture capital

## Abstract

**Background:**

We consider two key challenges that early-stage biotechnology firms face in developing a sustainable financing strategy and a sustainable business model: developing a valuation model for drug compounds, and choosing an appropriate operating model and corporate structure. We use the specific example of Unravel Biosciences—a therapeutics platform company that identifies novel drug targets through off-target mechanisms of existing drugs and then develops optimized new molecules—throughout the paper and explore a specific scenario of drug repurposing for rare genetic diseases.

**Results:**

The first challenge consists of producing a realistic financial valuation of a potential rare disease repurposed drug compound, in this case targeting Rett syndrome. More generally, we develop a framework to value a portfolio of pairwise correlated rare disease compounds in early-stage development and quantify its risk profile. We estimate the probability of a negative return to be $80.8\%$ for a single compound and $56.1\%$ for a portfolio of 8 drugs. The probability of selling the project at a loss decreases from $79.2\%$ (phase 3) for a single compound to $55.4\%$ (phase 3) for the 8-drug portfolio. For the second challenge, we find that the choice of operating model and corporate structure is crucial for early-stage biotech startups and illustrate this point with three concrete examples.

**Conclusions:**

Repurposing existing compounds offers important advantages that could help early-stage biotech startups better align their business and financing issues with their scientific and medical objectives, enter a space that is not occupied by large pharmaceutical companies, and accelerate the validation of their drug development platform.

## Background

Rare or orphan diseases are defined by the Orphan Drug Act of 1983 [1] as conditions that affect fewer than 200,000 individuals in the $\text{United States (U.S.)}$. While any single orphan disease affects only a small number of individuals, in aggregate there are more patients suffering from a rare disease than from more common ones. With more than 7000 rare diseases identified today, the total number of Americans living with a rare disease is estimated at 25–30 million [2, 3]. In comparison, 19 million new cancer cases are detected each year around the world; in 2021, 1.9 million individuals were diagnosed with cancer in the U.S [4].

The Orphan Drug Act was set up to mitigate the challenges of drug development for rare diseases—including delays in diagnosis due to a lack of public awareness and medical expertise—through incentives such as tax benefits, a 7-year minimum period of market exclusivity, and waivers of Prescription Drug User Fee Act (PDUFA) fees [5]. Nevertheless, large pharmaceutical companies are still more focused on diseases with much larger patient populations, leaving early-stage biotechnology companies as key participants in the research and development (R&D) process for rare diseases [6, 7]. As a result, financing drug development for rare diseases has been a major challenge.

A potential solution that addresses the rising cost and low probability of success (PoS) of developing new drugs has emerged over the past two decades. Drug repurposing, also known as drug repositioning [8], consists of identifying new indications for existing, abandoned, or shelved drugs, or for candidates under development [9–11]. Drug repurposing is viewed as an attractive way to lower development costs, shorten development time, increase the $\text{PoS}$, and maximize the potential impact of drug compounds for a broader population of patients [12, 13]. In fact, around one-third of new drugs and vaccines approved by the U.S. Food and Drug Administration (FDA) come from drug repurposing [12].

In this paper, we turn our attention to Unravel Biosciences,^1^ an early-stage therapeutics venture developing new therapeutics for genetic diseases by leveraging proprietary artificial intelligence (AI) models of patient RNA networks for clinical stratification and discovery of new targets that can be tested using existing drugs. The computational engine is coupled with on-demand genetic disease model generation and whole animal screening. Unravel Biosciences combines target-agnostic computational drug prediction with cognitive and behavioral screening to accelerate and de-risk effective treatments for patients living with rare genetic diseases and combine rare patient groups into larger, more commercially-attractive markets.

In particular, Unravel Biosciences has already generated three lead compounds for Rett syndrome, two of which fall into the category of repurposed orphan drugs, should they be commercialized. Rett syndrome is a rare genetic neurological disorder that occurs almost exclusively in girls and leads to severe impairment of their ability to speak, walk, eat, or breathe easily [14]. Using Unravel’s Rett syndrome compounds as a case study for situations in which repurposing through drug reformulation may be commercially attractive, we develop a detailed valuation framework to estimate the financial value of a rare disease drug compound. This calculation involves the careful consideration of $\text{PoS}$ [10, 15–18] and the development costs [10, 11, 19–21] of repurposed drugs for rare diseases.

Applying concepts from classical portfolio theory [22–25], it is also possible to improve the risk profile of a drug development project by considering the development of a portfolio of drugs instead of a single drug compound [26–28]. Rare diseases are generally believed to present low pairwise correlations in part due to their inherent nature as monogenic disorders [5, 29, 30], although this analysis can still be performed when the drugs in a portfolio are pairwise correlated. In this paper, we apply the diversification techniques developed in [5, 26, 29] to determine the value and risk profile of a hypothetical portfolio of repurposed compounds for rare diseases suggested by Unravel Biosciences.

The objective of this paper is to address two key challenges faced by early-stage biotech startups in the space of drug repurposing for rare diseases. Using Unravel Biosciences as a case study, we first develop a sustainable financial valuation model for drug repurposing (for a single drug compound as well as for a portfolio of moderately correlated compounds). Secondly, we explore different operating models and corporate structures commonly used by early-stage biotech startups and discuss their advantages and disadvantages in the context of drug repurposing.

In the next section, we present the assumptions in our valuations of the Rett syndrome compound as well as the portfolio of rare disease compounds. We then discuss and contrast the results obtained for both valuations before turning to a survey of key operating models and corporate structures observed among early-stage biotech companies.

## Methods

We begin our analysis by describing the procedure used to produce financial valuations for the Rett syndrome drug compounds and for a portfolio of drug compounds for rare diseases.

### Valuation of Rett syndrome compounds

Unravel Biosciences has proposed two lead compounds to treat Rett syndrome. Because these compounds qualify as repurposed compounds, Unravel intends to follow the 505(b)(2) path toward FDA approval [31], which gives them the ability to use previously completed research as part of their FDA application, an approach that is typically cheaper and faster than following a traditional $\text{new drug application (NDA)}$ path (the 505(b)(1) path) or an $\text{abbreviated NDA}$ path (the 505(j) path). We therefore consolidate all preclinical development through phase 1 into a single phase. We describe and discuss the assumptions of the single-compound valuation model next.

#### Data and assumptions

The base case of our analysis is calibrated using widely accepted sets of parameters for the drug development process and the commercial stage that follows upon FDA approval  (see Tables 1 and 2). We also provide adequate lower and upper bounds for these parameters to ensure the robustness of our results.

**Table 1:** Development stage assumptions used for the Rett syndrome compound valuation

(a) Probabilities of success, PoS	Lower bound	Base case	Upper bound	Prob. of arriving to phase
Discovery, preclinical development, and phase 1	62%	67%	72%	100%
Phase 2	43%	53%	63%	67%
Phase 3	59%	69%	79%	36%
FDA submission-to-launch	94%	94%	94%	25%
Total cumulative probability of success (PoS)	15%	23%	34%	–

(b) Time for development (years)	Lower bound	Base case	Upper bound	
Discovery, preclinical development, and phase 1	1	1	1	
Phase 2	1	2	3	
Phase 3	1	2	3	
FDA submission-to-launch	1	1	1	
Total	4	6	8	

(c) Cost of development ($, in millions)	Lower bound	Base case	Upper bound	
Discovery, preclinical development, and phase 1	1	4	7	
Phase 2	9	14	40	
Phase 3	22	35	99	
FDA submission-to-launch	37	37	37	
Total	69	90	183

**Table 2:** Commercial stage assumptions used for the Rett syndrome compound valuation

(a) Price and adoption rate assumptions	Lower bound	Base case	Upper bound				
Annual price ($)	30,000	40,000	50,000				
Adoption rate (peak, segment of 0–12 years)	35%	50%	65%				

(b) Commercial stage assumptions							
Period of exclusivity (U.S.)	14.0						
Cost of Goods Sold (COGS, % of sales)	10%						
Selling, General, and Administrative Expenses (SG&A, % of sales)	30%						
Research and Development (R&D, % of sales)	5%						
SG&A as % of R&D at pre-revenue stage	40%						
Tax rate	20%						
*Additional benefits*							
Patent term (in years)	20						
Orphan drug exclusivity (in years)	7						
Likelihood/strength of IP protection	100%						
Pediatric exclusivity (in years)	0.5						
Tax credit on R&D (up to Phase 3)	25%						
Priority Review Voucher (pediatric designation) valuation ($, in millions)	100						
Year in which the Priority Review voucher is granted (second year of Phase 2)	3						
*Financial assumptions*							
Discount rate	$$13\%$$						

(c) Patient population by age (U.S.)	Women in U.S. (in thousands)	Rett syndrome prevalence (per 1000)	Total patients				
0 to 12 years	25,770	0.125	3221				
12 to 24 years	25,313	0.120	3038				
24 to 48 years	51,749	0.115	5951				
48 to 85 years	64,090	0.060	3845				
Total	166,922	0.096	16,055				

(d) Adoption rates by age group per year following FDA approval (base case)	1	2	3	4	5	6	7
0–12 years	16.67%	33.33%	50.00%	50.00%	50.00%	50.00%	50.00%
12–24 years	12.50%	25.00%	37.50%	37.50%	37.50%	37.50%	37.50%
24–48 years	8.33%	16.67%	25.00%	25.00%	25.00%	25.00%	25.00%
48–85 years	0.00%	0.00%	0.00%	0.00%	0.00%	0.00%	0.00%
Total number of patients	1412	2825	4237	4237	4237	4237	4237
Peak year	Year 3 (following FDA approval)						

*Probabilities of Success per Phase,* $\textit{PoS}$. The $\text{PoS}$ is a key assumption of any drug compound valuation model. We average the published phase-by-phase $\text{PoS}$ estimates found in [15–17] for rare diseases, as well as estimates of $\text{PoS}$ found in [18] for orphan drugs, and in [10] for repurposed drugs. We obtain the $\text{PoS}$s per phase displayed in Table 1, and calculate a total cumulative $\text{PoS}$ of 23.0% in the base case. This value aligns with the 22–25% estimated in most orphan drug studies [18]. We select the lower (upper) bound estimate of 14.8% (33.7%) by reducing (increasing) the $\text{PoS}$ for the combined discovery, preclinical, and phase 1 development by $5$ percentage points, reducing (increasing) the $\text{PoS}$ for phase 2 and 3 by $10$ percentage points, and leaving the $\text{PoS}$ for FDA approval unchanged. These estimates are consistent with the most pessimistic scenarios for orphan drugs and the most optimistic scenarios for repurposed drugs recorded in the literature [10, 15–18]. We provide a more granular view of the assumptions made in Table 1(a).

*Development Costs.* We estimate the development costs using analyses of key cost drivers for pharmaceutical trials in the U.S. [19], studies about the cost of pivotal trials for novel therapeutic agents approved by the FDA in 2015–2016 [20], comparisons between cost estimates for de novo and repurposed drugs [10, 11], and comparisons of the cost of clinical trials for orphan and non-orphan drugs [21]. The baseline total out-of-pocket development costs are estimated by averaging the cost occurring at each phase across the different data sources. In addition, for better consistency across data sources when estimating upper and lower bounds, phase 3 costs are obtained by averaging the ratio between phase 3 and phase 2 costs across the data sources and multiplying this ratio by the baseline phase 2 cost estimated previously. Finally, orphan drug designation allows us to deduct the $\$2.9$ million PDUFA fee waiver [32] from the $\$40$ million FDA submission-to-launch cost estimated in [33]. In the base case, we estimate total out-of-pocket costs of development of $\$90$ million. Lower and upper bounds are estimated to be $\$69$ million and $\$183$ million, respectively, based on the same procedure as for the baseline case. We provide a more granular view of the assumptions made in Table 1(c).

*Time for Development*. The timeline of the drug development process for repurposed drugs can be estimated using ClinicalTrials.gov and [10, 15–18]. We estimate an average duration of 1 year for phase 1, 2 years for phase 2, 2 years for phase 3, and 1 year for FDA submission-to-launch and approval for a total duration of 6 years. We estimate an upper (lower) bound on the duration by averaging the higher (lower) time for development for repurposed drugs. In addition, we consider the longest and shortest durations observed in studies from ClinicalTrials.gov for Rett Syndrome [34, 35] when estimating the lower bound for the clinical trial durations. The lower and upper bounds are set at 4 and 8 years, respectively. We provide a more granular view of the assumptions made in Table 1(b). For greater flexibility, we assume that costs are distributed uniformly through time in each phase, and calculate the $\text{risk-adjusted net present value (rNPV)}$. This allows us to generalize our timeline to non-integer time values (for example, our framework would still work if we were to assume 2.75 years of development time in phase 2).

Furthermore, we assume a pairwise correlation of 0.5 for the time and cost of development between phases 2 and 3. We model this correlation across phases using the Gaussian copula methodology developed in [5, 26–29].

*Patient Adoption and Price.* As outlined in Table 2, we assume a staged adoption rate across different age ranges within the patient pool in the U.S. In general, assumptions about patient adoption need to be refined through conversations with the company and clinical contacts interested in the development of the drug. For the purposes of our analysis, we assume the adoption rate will peak at 50% of patients in the 0–12 years age group in year 3 following FDA approval in the base case, with the lower and upper bounds set to 35% and 65%, respectively. We further assume adoption rates in years 1 and 2 will be one-third and two-thirds of the peak, respectively, and that the adoption rates for the 12–24 years and 24–48 years age groups will be 75% and 50% of the adoption rate for the 0–12 years age group, respectively. Based on these assumptions, we reach a peak of 4237 patients in the third year following FDA approval in our base case (with the lower and upper bounds set at 2996 and 5509 patients, respectively).

In this example, we use an average list price of $\$40{,}000$ per year per patient, based on the price of other anti-epileptic drugs targeting other specific diseases (e.g., Epidiolex (cannibidiol), used to treat Dravet syndrome and Lennox-Gastaut syndrome, priced around $\$32{,}500$ per year, and Onfi (clobazam), used to treat Lennox-Gastaut syndrome, priced around $\$30{,}000$ per year). The lower and upper bounds for the list price are set at $\$30{,}000$ and $\$50{,}000$ per year, respectively. The model price assumption could be further refined through a multi-factor analysis of the pricing of recent treatments launched to market within the $\text{central nervous system}$ rare diseases space: for example, the proposed price might be negatively correlated with the total prevalence and the potential of receiving multiple indications, and positively correlated with the severity of the disease and the impact of the medication. Furthermore, existing treatments for the same disease could act as an anchor for the price. Ultimately, the factors used in such an analysis should be tailored to the drug and indication considered. However, we do not recommend using $\text{quality-adjusted life year (QALY)}$ analyses within the rare diseases space to estimate the potential price of a compound, as $\text{QALY}$ thresholds can vary significantly across time and institutions. For example, in the $\text{United Kingdom}$, the National Institute for Health and Care Excellence uses a threshold up to $\pounds 300,000$ for rare diseases (vs. its standard threshold of $\pounds$20,000–$\pounds$30,000) [36].

Finally, we should note that as compounds for Rett syndrome are brought to market, e.g., trofinetide (approved March 2023 as Daybue, Acadia Pharmaceuticals, $375,000/year), their price would likely be a reasonable estimate of the upper bound for the potential price of Unravel’s compound. This type of ceiling estimation has been used in the past, for example, with Cerezyme (imiglucerase) for Gaucher’s disease [37], Orkambi (lumacaftor/ivacaftor) for cystic fibrosis [38], and Esbriet (pirfenidone) for pulmonary fibrosis [39]. Nevertheless, we have opted for a more conservative price point in line with other orphan drugs despite Daybue's price due to its very limited market exposure as of publication.

*Other Commercial Stage and Financial Hypotheses.* The typical discount rates used for $\text{rNPV}$ analyses range from 10–13%. A discount rate is commonly used in financial valuations to reflect the time value of money: a higher discount rate implies less patient investors or riskier projects (see [40] for a more in-depth discussion). We opt for a $13\%$ discount rate to obtain a lower-bound estimate of the rNPV. Using smaller values for the discount rates would significantly increase the $\text{rNPV}$ (as will be shown by decreasing the discount rate to $r=11\%$). We also calibrate other commercial-stage assumptions such as the $\text{cost of goods sold (COGS)}$ and selling, general, and administrative expenses (SG&A) against reference values used in the space of rare neurological disorders [41, pp. 78–79].

$\textit{Intellectual Property (IP)}$ *Protection, Exclusivity Period, and Other Benefits.* In this analysis, we assume a period of $20$ years for patent protection of the repurposed compound starting at the beginning of the discovery/preclinical development phase, which is typical in the U.S. Under the baseline scenario considered, given 6 years of clinical development until the drug receives FDA approval, we obtain a period of exclusivity of 14 years.

Given the type of compound and disease targeted, if this assumption did not hold, a rare disease compound may yet still be eligible to receive the orphan drug, pediatric exclusivity, and/or rare pediatric disease  designation. Orphan drug designation grants a minimum exclusivity period of 7 years (as was the case with trofinetide for Rett syndrome) [1]. In addition, the drug could potentially benefit from an additional 6 months of exclusivity with the pediatric exclusivity designation [42]. With rare pediatric disease designation, the company could also obtain a priority review voucher upon regulatory approval, which would provide an additional source of value (in 2019, priority review vouchers were sold between $\$80$ million and $\$130$ million) [43]. We do not include these additions in the valuation of our compound so as to obtain a more conservative $\text{rNPV}$, however, for comparison purposes, we do conduct a separate valuation that assumes a priority review voucher is obtained.

#### Valuation model: multi-stage Monte Carlo simulation

In addition to performing a sensitivity analysis, we test the robustness of our results by running a multi-stage Monte Carlo simulation of our financial valuation using the @modelrisk software. In this simulation, we test the same assumptions as in our sensitivity analysis, proceeding as follows.

*Development Costs.* Development costs are assumed to follow a PERT distribution using the lower-bound estimate ($\$69$ million) as the minimum parameter, the base case estimate ($\$90$ million) as the most likely parameter, and the upper bound estimate ($\$183$ million) as the maximum parameter.

*Time for Development.* The development timeline is also assumed to follow a PERT distribution ranging between 4 and 8 years (with a mode of 6 years).

*Patient Adoption and Price.* The annual price is assumed to follow a normal distribution centered at $\$40{,}000$, which is calibrated such that its $95\%$ confidence interval ranges between $\$30{,}000$ and $\$50{,}000$. We also assume the peak adoption rate will follow a normal distribution in its youngest age range. Its $95\%$ confidence interval is calibrated to match our lower and upper bound peak adoption rate estimates.

We model the drug development process as a sequence of Bernoulli trials using our previously defined $\text{PoS}$ for each phase. The duration of each phase follows the PERT distribution described above. We run 10,000 Monte Carlo simulations to estimate the value of the drug compound upon completion of each phase, the value of the drug compound upon the successful completion of each phase, and the net present value (NPV) in year 0 of selling the drug compound at the end of each phase, priced at its $\text{rNPV}$ at the end of the phase.

### Portfolio valuation

As discussed earlier, considering a portfolio of drugs can potentially lower the investor’s risk through diversification. Here, we explore the financial performance of different portfolios of drugs within the space of rare diseases, and run Monte Carlo simulations using the @modelrisk software as described in the previous section. The portfolio of drugs under consideration is based on recommendations received from Unravel Biosciences.

#### Data and assumptions: development stage and valuation hypotheses

We summarize in Table 3 the main assumptions used to calibrate the parameters of our portfolio simulations. Commercial stage assumptions are the same as those used for the Rett syndrome compound valuation (see Table 2). The lower and upper bounds provided are used to test the robustness of our results. We discuss our assumptions regarding the development and commercial stages of the drugs composing our portfolio in Appendix A.

**Table 3:** Development stage assumptions used for the portfolio of rare disease compounds and the probability distributions used to perturb our assumptions

		Normal case		"Me-too" case	
(a) Probabilities of success, PoS	Distribution	PoS	Prob. of arriving to phase	PoS	Prob. of arriving to phase
Discovery, preclinical development, and phase 1	Bernoulli	67%	100%	72%	100%
Phase 2	Bernoulli	53%	67%	63%	72%
Phase 3	Bernoulli	69%	36%	79%	45%
FDA submission-to-launch	Bernoulli	94%	25%	94%	36%
Total cumulative PoS		100%	23%	100%	34%

(b) Time for development (years)	Distribution	Lower bound	Base case	Upper bound	
Discovery, preclinical development, and phase 1	PERT	1	1	1	
Phase 2	PERT	1	2	3	
Phase 3	PERT	1	2	3	
FDA submission-to-launch	PERT	1	1	1	
Total		4	6	8	

(c) Cost of development ($, in millions)	Distribution	Lower bound	Base case	Upper bound	
Discovery, preclinical development, and phase 1	PERT	1	4	7	
Phase 2	PERT	9	14	40	
Phase 3	PERT	22	35	99	
FDA submission-to-launch	PERT	37	37	37	
Total		69	90	183	

We consider drugs that are and that are not a “follow-on” of other drugs for the same disease that have been proven to be effective and reached phase 2 (i.e., "me-too" drugs)

### Operating models and corporate structures for biotech startups

While developing a realistic financial valuation framework of a potential portfolio of rare disease drug compounds is a crucial first step, creating a sustainable financial model for drug repurposing for rare diseases also requires thinking about the company itself and its structure.

We address this second challenge by exploring potential operating models and corporate structures commonly found across early-stage biotech startups, based on domain knowledge from the authors and from Unravel Biosciences. In this analysis, we confine our attention to three common operating models and one key corporate structure. As an operating model, the startup can explore technology licensing, discovery price provision, and the sale or out-licensing of assets after phase 2.^2^ We then consider the creation of subsidiaries, a common choice of corporate structure among early-stage biotech companies. A more in-depth study of operating models and corporate structures is available in Appendices B and C.

## Results

We first present the results of our simulations for the valuation of the Rett syndrome compounds before turning to the portfolio of compounds, and then explore different operating models and corporate structures.

### Valuation of Rett syndrome compounds

**Fig. 1:**
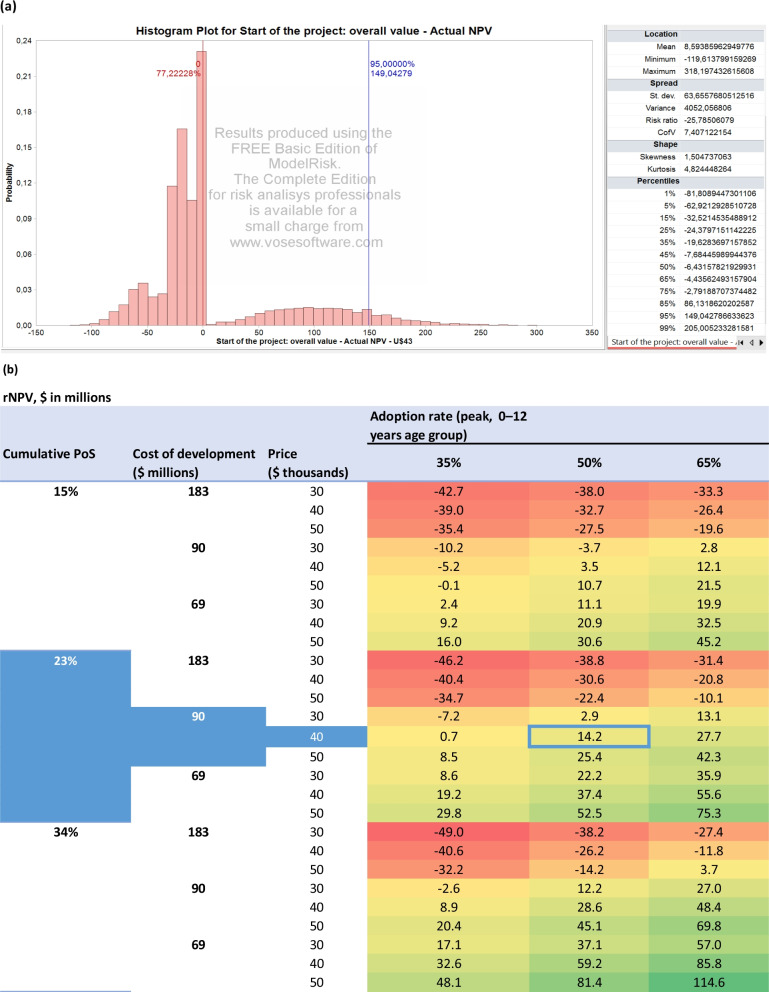
Summary of results (**a**) and sensitivity analysis (**b**) for the Rett syndrome compound. In panel (**a**), actual NPVs obtained by running 10,000 Monte Carlo simulation paths in ModelRisk using base-case parameters. In panel (**b**), blue highlighting indicates the base-case parameters, and heat-map coloring indicates the impact on the estimated rNPV (with lower values colored in red and higher values colored in green)

We summarize the results for the valuation of the Rett syndrome compounds in Fig. 1 and Table 4. For comparison, the corresponding results obtained using a discount rate of $r=11\%$ are presented in Table 5. Using the financial model described in the Methods section, we obtain an overall $\text{rNPV}$ of $\$14.2$ million at the start of the project for the Rett syndrome compound in the U.S market. This estimate assumes a cumulative $\text{PoS}$ of 23.0%, a $\$90$ million cost of development, a list price of $\$40{,}000$, and a peak adoption rate of $50\%$ among children between 0 and 12 years old three years after the FDA approval of the drug. Taking into account a $25\%$ tax credit for R&D spending up to phase 3, the estimated $\text{rNPV}$ increases to $\$18.6$ million. Furthermore, obtaining a priority review voucher would increase the estimate to an $\text{rNPV}$ of $\$46.4$ million.

**Table 4:** Summary of results of the Monte Carlo simulation for the Rett syndrome case using a discount rate of $r=13\%$

		Monte Carlo simulation		
	Expected rNPV after each phase	Average rNPV	Standard deviation of rNPV	Upper bound on the rNPV
Discovery, preclinical development, and phase 1	32.3	23.19	21.45	123.98
Phase 2	117.1	105.07	48.10	325.33
Phase 3	292.3	288.37	80.15	687.25
FDA submission-to-launch	406.5	401.78	96.35	881.26
Overall	14.2	8.79	12.81	69.20

All $\text{rNPV}$s are expressed in millions of dollars

**Table 5:** Summary of results of the Monte Carlo simulation for the Rett syndrome case using a discount rate of $r=11\%$

		Monte Carlo simulation		
	Expected rNPV after each phase	Average rNPV	Standard deviation of rNPV	Upper bound on the rNPV
Discovery, preclinical development, and phase 1	46.9	37.40	25.32	138.80
Phase 2	148.0	135.62	55.47	374.56
Phase 3	339.2	334.72	91.10	716.11
FDA submission-to-launch	455.7	450.36	107.57	900.73
Overall	23.3	17.52	15.34	80.06

All $\text{rNPV}$s are expressed in millions of dollars

**Fig. 2:**
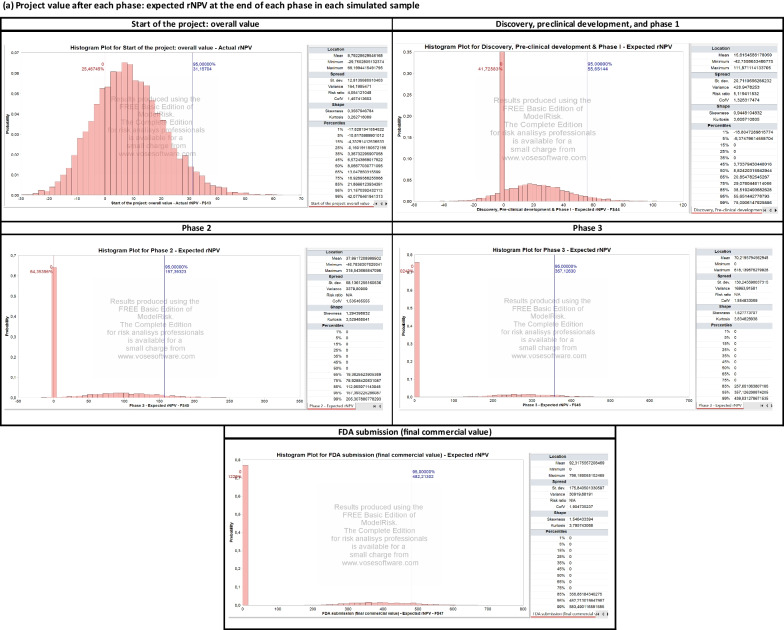

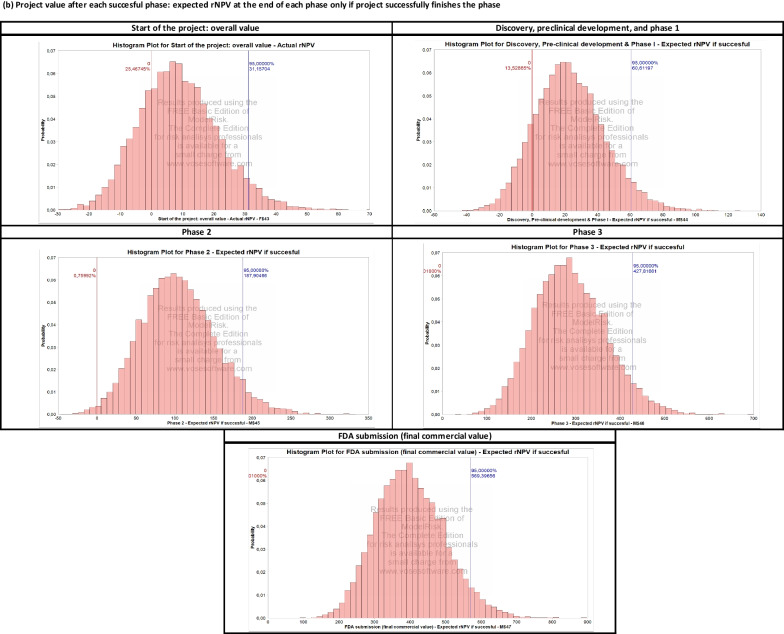

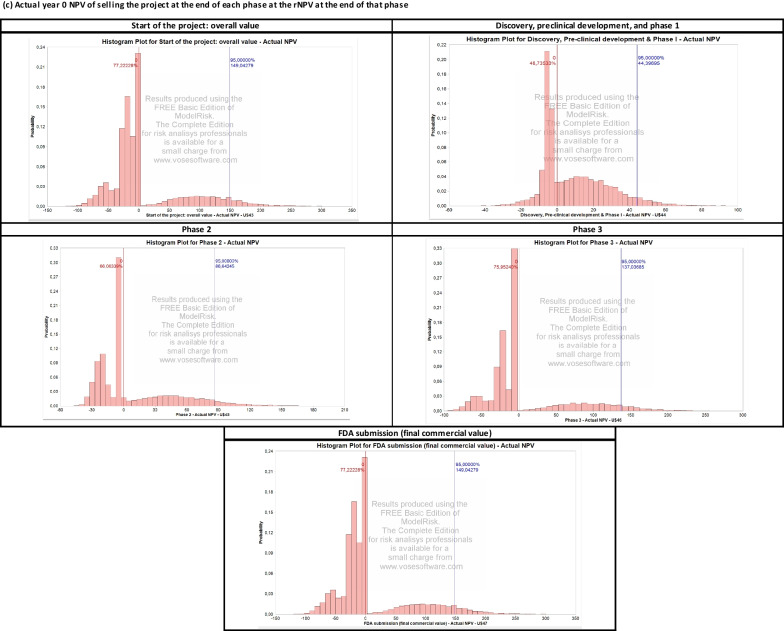
Summary of results of the Monte Carlo simulation for the Rett syndrome case

Perturbing the development and commercial stage assumptions over 10,000 Monte Carlo simulations yields the output presented in Fig. 2 and Table 4. The results are organized into the following three categories.

*Project Value Upon Completion of Each Phase.* A histogram summarizes the distribution of the expected $\text{rNPV}$ obtained at the end of each phase, successful or unsuccessful, in each simulated sample.

*Project Value Upon the Successful Completion of Each Phase.* Here, we consider only the projects that successfully complete the considered phase and plot the distribution of the expected $\text{rNPV}$ obtained at the end of that phase. These distributions are also used to calibrate the parameters of the portfolio of drugs simulated in the next section. In particular, we use the average, standard deviation, and $95\%$ confidence interval of the $\text{rNPV}$ distribution to calibrate the log-normal distribution of a drug in the portfolio.

*Actual NPV (in Year 0) of Selling the Project Upon Completion of Each Phase.* This discounts the $\text{rNPV}$ obtained at the end of each phase to year 0. The probability of a positive NPV in year 0 converges to a value similar to the cumulative $\text{PoS}$ used for each phase.

As shown in Fig. 2, a single Rett syndrome compound presents important financial risks. Using Monte Carlo simulations, we estimate an $80.8\%$ probability of observing a negative overall NPV, as well as a $38.3\%$, $67.5\%$, and $79.2\%$ probability of selling the project at a loss for phases 1, 2, and 3, respectively. Next, we investigate a classical way to reduce these risks by constructing a portfolio of rare disease compounds and discuss the potential impact of the operating model and corporate structure chosen by the biotech startup.

### Portfolio valuation of rare disease compounds

**Fig. 3:**
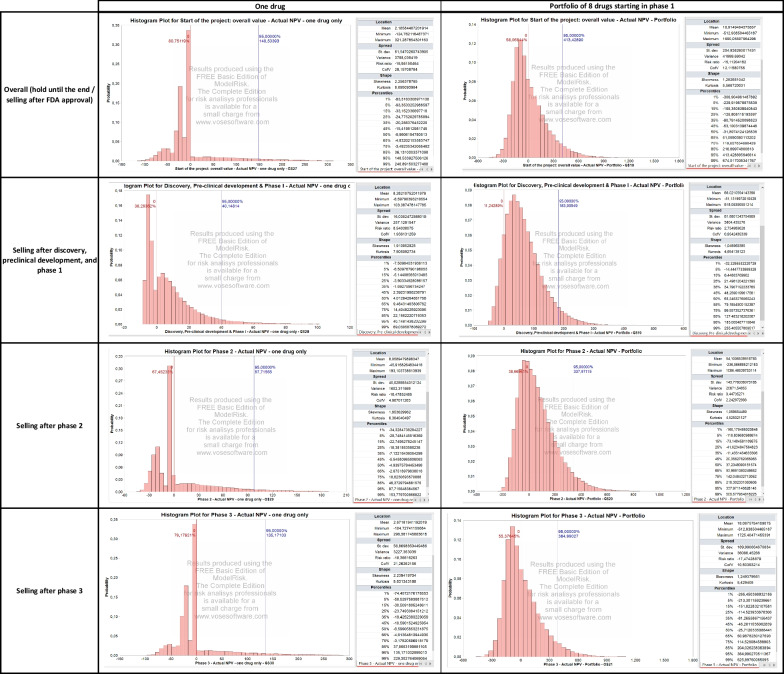
Results of the Monte Carlo simulation model for the portfolio of orphan drug compounds (right column) and for a single drug compound (left column) using a discount rate of $r=13\%$. We present the overall NPV of the project, as well as the NPV in year 0 of selling the project in phase 2 and in phase 3

**Fig. 4:**
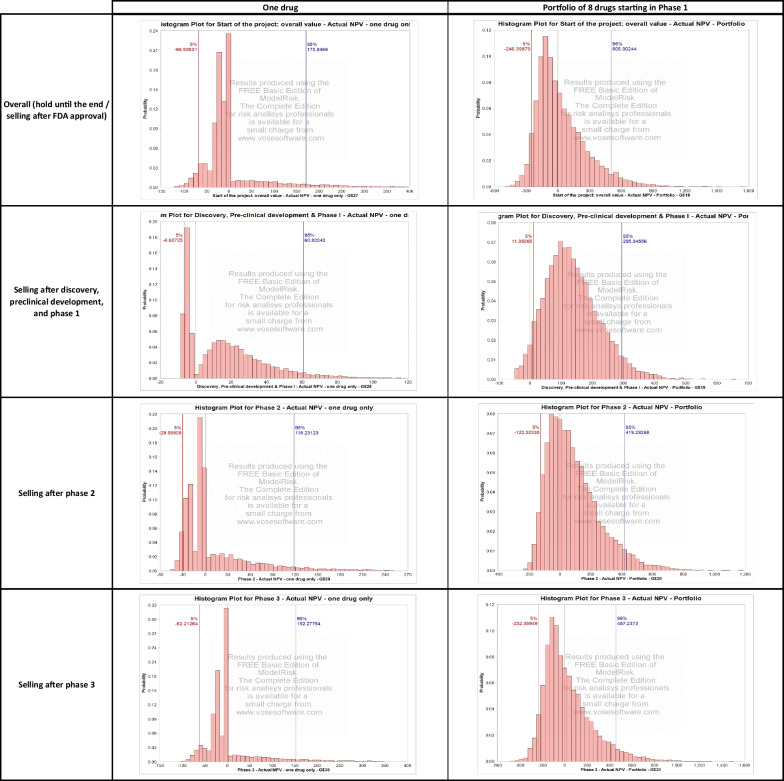
Results of the Monte Carlo simulation model for the portfolio of orphan drug compounds (right column) and for a single drug compound (left column) using a discount rate of $r=11\%$. We present the overall NPV of the project, as well as the NPV in year 0 of selling the project in phase 2 and in phase 3

To quantify the advantages of diversification in combining multiple orphan drug compounds into a single portfolio, we consider a portfolio of 8 drugs in the preclinical development phase modeled on the Rett syndrome drug compound described earlier, which follow the pairwise correlation structure described in Appendix A. In the same spirit as in the Rett syndrome simulation, we present in Fig. 3 the impact of perturbing the development and commercial stage assumptions to estimate the value of a portfolio of rare disease compounds over 100,000 Monte Carlo simulations. The corresponding results using a discount rate of $r=11\%$ are shown in Fig. 4. The results are organized into the following two categories.

*Combined Value of All Drugs in the Portfolio Upon Completion of Each Phase.* A histogram summarizes the distribution of the expected $\text{rNPV}$ of the portfolio of compounds at the end of each phase (successful or unsuccessful) in each simulated sample. Although in practice the drugs may not complete each phase simultaneously, we ignore these timing differences when calculating the aggregate $\text{rNPV}$.

*Total NPV (in Year 0) of Selling All Drugs in the Portfolio Upon Completion of Each Phase*. As described in the case of the Rett syndrome compound valuation, we discount to year 0 the overall $\text{rNPV}$ obtained at the end of each phase. This metric is particularly helpful in assessing and comparing the magnitude of financial risk across various portfolio structures. In fact, relying on present values is often preferred to using the return on equity, as the latter depends on additional external variables such as the portfolio’s capital structure and cost of capital.

The valuation results for a portfolio of repurposed orphan drug compounds are summarized in Fig. 3. We find that a portfolio of 8 drugs has a probability of generating a negative overall NPV of $56.1\%$. The probability of selling the project at a loss is estimated at $11.2\%$, $38.7\%$, and $55.4\%$ for phases 1, 2, and 3, respectively.

### Selecting appropriate operating models and corporate structures for biotech startups

Early-stage biotech companies such as Unravel Biosciences often seek multiple sources of revenue streams to finance their R&D and establish their reputation while remaining competitive. While repurposing drugs can be profitable, as shown in the previous section  with a portfolio of 8 drugs, the fate of the biotech startup also hinges critically on the operating model and corporate structure selected. In this section, we explore the advantages and disadvantages of three typical operating models within the biotech world, and discuss one common choice of corporate structure among early-stage biotech companies, which entails creating subsidiaries. We consider the following three main revenue streams: technology licensing, discovery service provision, and the sale or out-licensing of assets after phase 2. A summary can be found in Table 6, and a more detailed discussion of operating models and corporate structures can be found in Appendices B and C.

**Table 6:** Survey of common operating models used in the biotech startup world

Operating model	Revenue stream	Pros	Cons
Technology Licensing	Licensing fee paid upfront	Limited upfront investment (lower risk)	Reduced differentiation from other biotech companies, which leads to a reduced potential of completing a licensing agreement with a big pharma
	Fee for service revenue	Generates revenues early, making it easier to attract funding from venture capital firms	Reduced negotiation power due to higher clinical development risk
	Near-term preclinical milestone payments	Builds credibility before expanding downstream into development and commercialization	Reduced long-term upside due to potential replication risks
	Royalty payments on future sales of marketed products(typically a small percentage of sales)		
Discovery Service Provision	Licensing fee paid upfront	Build working relationships with large pharmaceutical companies, paving the way for a more profitable partnership in the future	Limits the startup to discovery service provision (the startup cannot venture with development if it finds a promising asset)
	Fee for service revenue	Provides revenue and cash flow visibility	Gives up all the upside to the large pharma partner
	Discovery milestone payments for discovered drugs approved o by the pharmaceutical company	Leverage the big pharma’s expertise in late- o stage development and commercialization while sharing the risk	
	Royalties on drug sales (larger than for technology licensing)	Modest revenues generated by upfront fee and milestone payments (in case the drug is not successful)	
Sale and Out-Licensing of Assets (after Phase 2)	Large upfront fee per asset o (significantly larger than technology licensing)	Higher return on investment compared to technology licensing	Higher upfront investment
	Large milestones payments for late-stage development and commercialization	Differentiates the startup from other competing technologies by offering a tested drug	Higher risk ahead of commercialization
	Royalties on drug sales (larger than for technology licensing)	Leverages the big pharma’s expertise in late-stage development and commercialization while sharing the risk	
		Modest revenues generated by upfront fee and milestone payments (in case the drug is not successful)	

We emphasize their relevance to biotech startups using the example of Unravel Biosciences and its drug repurposing opportunity as a concrete illustration, but the operating models and corporate structure covered here are applicable to a large range of biotech startups. In particular, although biotech startups aim to successfully develop new drug compounds, repurposing existing compounds can potentially provide additional advantages in early development stages of the company (by entering a space that is not occupied by large pharmaceutical companies and as a path to accelerating the validation of the drug development platform), especially when following a portfolio approach as well as the recommendations explored throughout this section and in Appendices B and  C.

#### Technology licensing

In the case of Unravel, large pharma companies could purchase licenses to gain access to the firm’s $\text{AI}$ repurposing tool, allowing them to develop new indications for their existing drugs. The large pharma companies would then go through all later stages of development independently, without support from the biotech startup.

*Revenue Streams*. Revenue streams from technology licensing typically include a licensing fee paid upfront (typically small for technology licensing); fee-for-service revenue (e.g., a yearly fee to use the startup’s technology); near-term preclinical milestone payments; and royalties on future sales of marketed products (which tend to be a small percentage of sales).

*Pros*. Technology licensing has the advantage of limited upfront financial investment, and therefore lower risk. Licensing is also a good way to start generating revenue, thus making it easier to attract funding from venture capital firms. Finally, it can help the startup build credibility before expanding downstream into development and commercialization.

*Cons.* Technology licensing also has certain disadvantages. It provides less differentiation from other biotech companies, and therefore lowers the chance of successfully completing a licensing agreement with big pharma companies. It also provides less negotiating power on the valuation of the startup’s technology, due to the significant risks in upcoming phases of clinical development. Finally, there will be less upside in the long run, as large pharmaceutical companies may be able to replicate the technology, potentially making the startup’s technology obsolete.

#### Discovery service provider

Similarly, the startup may be able to provide large pharma companies with drug discovery and early stage development services based on a fee-for-service contract. These services would target only the drugs of the pharma companies.

*Revenue Streams.* Revenue streams in the provision of discovery services typically include a licensing fee paid upfront; fee-for-service revenue (e.g., a yearly fee to use the startup’s technology); discovery milestone payments for discovered drugs approved by the pharmaceutical company; and royalties on drug sales (which tend to be significantly larger than in technology partnerships).

*Pros.* The startup may have the opportunity to build working relationships with large pharmaceutical companies, thus paving the way for a more profitable partnership in the future. In the meantime, the relationship provides revenue and cash flow visibility, both of which are important for early-stage startups. The startup would still leverage the large pharmaceutical company’s expertise in late-stage development and commercialization while sharing the risk. Finally, upfront fees and milestone payments would generate a modest return, in case the drug is not commercialized successfully.

*Cons.* The startup would be limited to discovery service provision, and would be unable to venture out to develop a promising asset. In effect, the startup would be giving all potential upside to its large pharma partner.

#### Selling and out-licensing assets after phase 2

Finally, the startup could decide to complete the initial discovery and development phases first before out-licensing or selling its assets to large pharmaceutical companies at the end of phase 2. In this scenario, the startup would spend 1–2 years in development until it obtains results from its phase 2 clinical trials, which would cost around $\$3{-}\$4$ million per drug in Unravel’s case. The startup would then either charge a sale fee (depending on the valuation of the Rett syndrome model) or an upfront licensing fee, collect milestone fees for the successful completion of phase 3 and an FDA approval, and collect royalties on product sales in the case of FDA approval.

*Revenue streams.* Revenue streams from the sale and out-licensing of assets typically include a large upfront fee per asset, which would tend to be significantly larger than those obtained through technology licensing, due to the higher investment by the startup and the increased odds of approval after positive phase 2 results. They would also include large milestone payments for late-stage development and commercialization, and royalties on drug sales, which are also usually larger than those obtained through a technology partnership.

*Pros.* On the positive side, the startup would obtain a higher return on investment compared to technology licensing, while differentiating itself from other competing technologies by offering a tested drug. The startup would still be able to leverage a large pharmaceutical company’s expertise in late-stage development and commercialization while sharing the risk. Finally, the upfront fee and milestone payments would generate a modest return in the case that the drug is not successfully commercialized.

*Cons.* The principal drawback of this approach is that it requires a heavier upfront investment, which significantly increases the risk ahead of commercialization.

#### Setting up subsidiaries, a common choice of corporate structure

A subsidiary refers to a company that is owned or is controlled by another company, sometimes referred to as the parent or holding company. The subsidiary is a separate entity from the parent company, having its own tax and regulatory treatments, as well as separate liabilities. This differentiates them from business divisions, which are held within the same company, and are not distinct legal entities.

There are several reasons for which a company might choose to set up a subsidiary instead of deciding to become a business division. As discussed above, a subsidiary is a good way to limit the liability of the parent company. This limitation is especially useful in risky endeavors like drug development, with high probabilities of large losses. It is also useful in the presence of external risks, such as regulatory restrictions or environmental hazards, similar to the risks borne by mining, oil, and gas companies.

Another benefit of setting up a subsidiary is that the new entity can focus on a specific area, which helps to attract new talent and generate specific knowledge in that domain. This can be particularly useful for biotech companies that are developing drugs in multiple therapeutic areas, allowing scientists to make use of their research with a specific focus on a particular disease or drug and providing a clear mission and area of focus for employees in general. In the long run, this can lead the subsidiary to generate domain-specific knowledge in a particular therapeutic area or disease, which can be useful in advancing the development of its treatments.

Similarly, having a subsidiary corporate structure may help to attract investors to the company that are interested in the specific focus area of a subsidiary, but do not want to invest in the parent company. In fact, public holding companies usually trade at a discount vis-à-vis their subsidiaries because of their business diversification—investors would rather invest in pure plays, and diversify their portfolio as they see fit, not as the holding company does. By setting up subsidiaries, it is easier (and often cheaper) for the holding company to sell or create a spin-off, as the company would only need to sell the shares of the subsidiary. Otherwise, it would need to decide whether to sell its assets, or place its assets into a new company and sell the new company. Likewise, by setting up different subsidiaries, a company can more easily place them into a special-purpose vehicle if it wishes to securitize its assets.

Lastly, for biotech companies, setting up subsidiaries can be very helpful when partnering with foundations or patient-advocacy groups if they are targeting a single drug or disease. Most of these entities offer grants and money to the development of treatments to the specific diseases mandated by their mission, making it easier for them to partner with a subsidiary working on their disease alone than with a company working on several unrelated diseases at once that would therefore need to differentiate its assets.

While the subsidiary corporate structure model has several advantages, it also presents some drawbacks that need to be considered when deciding on how to set up a company. These disadvantages include the legal and administrative requirements necessary to prepare separate tax filings, board meetings, and financial statements, which can be time-consuming and expensive and may require hiring a company that offers these services. Furthermore, separate tax filings may prevent the holding company from offsetting gains and losses from its different subsidiaries, which is commonly done when a company is structured as a business division. This does not hold if the holding company owns more than 80% of the shares in the subsidiary, but it still limits the holding company to raising capital to expand its subsidiary by selling shares.

Some companies enter into license agreements with partners or providers for their day-to-day operations. In the pharmaceutical industry, these license agreements can dictate the use of a certain drug or production technology. If different companies within a holding group (for example, a parent and subsidiary) or multiple subsidiaries need the same licenses, each company may need to enter into its own license agreement since they are separate legal entities.

While a crucial advantage of setting up a subsidiary is that it can limit the liability of the parent company, to maintain this protection, both companies need to keep transactions at arm’s length, which means that both companies must act as if they are independent and separate entities. However, arm’s-length transactions may limit the ability of a holding company to allocate resources among the different assets in development, which could be strenuous for a company that lacks excessive capital or funding, such as a startup.

Lastly, the subsidiary model limits the ability of the holding company to allocate resources between its different assets and reduces the benefit of buying or procuring services or supplies in bulk for all its different assets. This does not mean that the benefit of scale is lost, but rather that there is an extra layer that may complicate matters, especially if the subsidiary is not wholly owned by the parent company.

## Discussion

Using the example of the Rett syndrome compounds under development by Unravel Biosciences, we address two key challenges that early-stage biotechnology firms face while building their financial strategy: the development of a valuation framework for drug repurposing in the space of rare diseases (for a single compound as well as for a portfolio of moderately correlated rare disease compounds) and the choice of an appropriate operating model and corporate structure.

In this section, we first discuss the financial valuations described in the previous section. We then consider the impact of a biotech startup’s operating model and corporate structure through three concrete examples: Recombinetics, BridgeBio, and Recursion Pharmaceuticals. Finally, we conclude our discussion by addressing the limitations of our analysis.

### Financial valuations

Based on the multi-stage Monte Carlo simulations we performed for both a single rare disease compound and a portfolio of 8 moderately correlated rare disease compounds, we find the risk profile of a portfolio of drugs to be much more attractive than that of a single compound. In particular, the probability of observing a negative overall NPV, estimated for a single compound at $80.8\%$, decreases to $56.1\%$ for a portfolio of 8 drugs. Furthermore, the probability of selling the project at a loss, estimated for a single compound at $38.3\%$, $67.5\%$, and $79.2\%$ for phases 1, 2, and 3, respectively, decreases to $11.2\%$, $38.7\%$, and $55.4\%$ for phases 1, 2, and 3, respectively, when considering a portfolio of 8 drugs. The attractiveness of the portfolio relative to the single compound is robust to random perturbations in key development and commercial stage parameters, with a reduction in the risk of a negative overall $\text{rNPV}$ and a reduction in the probability of selling the project at a loss.

### Industry examples

By comparing the advantages and disadvantages of three typical operating models within the biotech world (technology licensing, discovery service provision, and the sale or out-licensing of assets after phase 2), and different choices of corporate structures that are currently popular among early-stage biotech companies, we have shown that it is imperative for a biotech startup to select an appropriate operating model and corporate structure. In this section, we provide three concrete examples of actual biotechnology companies that are either developing new drugs or working on repurposing existing drugs and discuss their business models.

*Recombinetics.* Recombinetics is a biotechnology company based in Minnesota focused on the use of gene editing to solve problems with organ transplants, evaluate preclinical drugs, and improve the genetic traits of farm animals. The company is structured around each of its subsidiaries, creating a new entity for each one. Regenevida develops transplantable cells, tissues, and organs using patient cells; Makana Therapeutics focuses on xenotransplantation by providing donor organs from genetically modified pigs; Surrogen evaluates preclinical drugs through the use of pig models of human diseases; and Acceligen is intended to improve the genetic traits of farm animals. Each subsidiary has its own team and has signed partnerships with different foundations (such as the Bill and Melinda Gates Foundation and Boston’s Brigham and Women’s Hospital), and opened up offices on different continents.

*BridgeBio.*^3^ BridgeBio is a company based in California that is focused on finding cures for genetic diseases. It has approximately 19 drugs under development in three different therapeutic areas. The company has been organized around its assets, with a subsidiary created for each drug. Each subsidiary is able to make its own decisions to hire employees, raise capital, and retain specific knowledge. Some of the companies are public, as is the parent company, which is able to leverage the portfolio approach at a holding company level. Similar to Recombinetics, drugs are still held in different subsidiaries even if they may fall within the same therapeutic area.

*Recursion Pharmaceuticals.* Recursion Pharmaceuticals, a biotech startup based in Utah, was founded in 2013 and focuses on drug repurposing. The company uses machine learning to analyze vast repositories of biological images to better understand how genes, proteins, and chemicals interact. As a result, the company can generate a set of hypotheses on whether existing drugs can be repurposed to target new diseases, creating a more efficient process for drug repurposing.

Recursion has established partnerships with various pharmaceutical companies in the short term to provide discovery services and out-license assets for drug development. One such collaboration was formed in 2016 with Sanofi Genzyme, which aimed to identify novel applications for Sanofi’s clinical stage molecules in numerous genetic disorders. Recursion also leverages its platform to assist pharmaceutical partners in their own drug discovery pipelines. In 2017, Recursion collaborated with Takeda Pharmaceutical to offer preclinical candidates for the latter’s development pipeline, using Recursion’s machine learning technology. As a result of the deal, Recursion is entitled to royalty payments on future sales of these drugs, if approved. Recursion also collaborated successfully with Bayer on cystic fibrosis research in 2020, and is currently running phase 2 trials for drugs that aim to treat cerebral cavernous malformation and neurofibromatosis of type 2. Since 2021, Recursion has been collaborating with Roche and Genentech to advance the drug discovery process in neuroscience and oncology by deploying the Recursion Operating System to identify novel biological relationships and initiate and advance therapeutic programs. Recursion received an upfront payment of $\$150$ million and is eligible for up to $\$300$ million in additional performance-based research milestones as well as tiered royalties on net sales. The company went public in April 2021 through an initial public offering that garnered $\$462.6$ million in net proceeds.

### Limitations of the analysis

Our findings must be qualified in several respects. First, for expositional simplicity, we assume an average list price of $\$40{,}000$ per year per patient as an input to the valuation model. In practice, we would expect the price of a repurposed drug to be smaller than or equal to the current price of the same drug used for another indication [44–46]. This means that the $\text{rNPV}$ obtained under the baseline case would be an upper bound on the $\text{rNPV}$ obtained if the average list price is lower than $\$40{,}000$, and a lower bound on the probability of selling the portfolio at a loss.

Second, we have made strong assumptions in this case study for illustrative purposes regarding the distributions of key parameters to the valuation model. For example, we assumed that development costs would follow a PERT distribution and the $\text{rNPV}$ obtained for a single drug compound follows a log-normal distribution. We encourage the reader to relax or modify these assumptions to better reflect their use case.

Finally, we have confined our attention to the case of Rett syndrome repurposed drug compounds. The same framework can be used to value a portfolio of other orphan diseases, but many parameters assumed in this model will need to be tailored to the application in mind. We strongly recommend leveraging domain expertise from researchers when calibrating the valuation model to a specific therapeutic area.

We believe that a nuanced consideration of these issues will be instructive in establishing a sustainable, realistic, and robust valuation of portfolios of repurposed drug compounds that is well suited to the biotech startup’s objectives. We hope that the simplicity and transparency of the framework proposed here can make it a potentially valuable tool when evaluating the viability of a potential portfolio of repurposed drug compounds for rare diseases.

## Conclusion

Repurposing existing compounds can potentially help biotech startups enter a space that is not occupied by large pharmaceutical companies and provide a path to accelerate the validation of its drug development platform. Although repurposing drugs can be profitable when following a portfolio approach, it is crucial to carefully select the appropriate operating model and corporate structure of the startup.

In this paper, we considered two challenges: the development of a valuation framework for drug repurposing in the space of rare diseases (for a single compound as well as for a portfolio of moderately correlated rare disease compounds), and the choice of an appropriate operating model and corporate structure for an early-stage biotech company. By addressing these two challenges in the specific context of an early-stage biotechnology company, we hope to illustrate the process by which business and financing issues can be better aligned with scientific and medical objectives. From a societal perspective, estimating the “fair” price of a drug can raise complex ethical debates. We hope that the framework developed here can also provide researchers, regulators, and practitioners with better tools to address these ethical questions and effectively align the incentives of every stakeholder.

## Figures and Tables

**Fig. 5:**
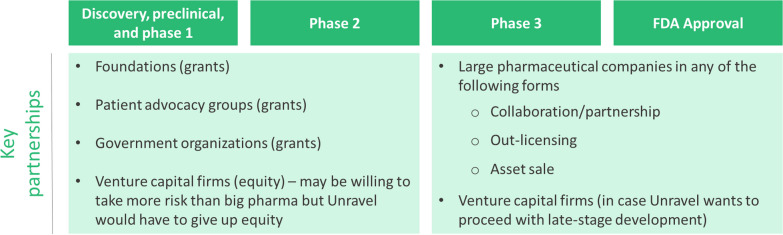
Key partnerships at different stages of asset development

## Data Availability

The Excel file designed during the current study is available from the corresponding author on reasonable request.

## Appendix A: Development stage and valuation hypotheses assumptions for a portfolio of drug compounds

In this section, we provide a more detailed description of the assumptions made for the financial valuation of a portfolio of 8 rare disease repurposed drug compounds.

### Development stage assumptions

For consistency, we use the assumptions made for the Rett syndrome compound and the perturbations made in the multi-stage Monte Carlo simulationas the base case. These parameters can be adjusted to align with other portfolios that may present different developmental aspects than the Rett syndrome compound previously considered.

We increase the flexibility of the model by accounting for more complex interactions between the drugs composing our portfolio. In particular, we allow some drugs in the portfolio to start in different phases of the drug development process. For example, some drugs may be a “follow-on” of other drugs for the same disease (i.e., “me-too” drugs), thus having a higher $\text{PoS}$ in the preclinical phase once the original drug has been proven to be effective and reaches phase 2. The $\text{PoS}$ of the drugs composing our portfolio are allowed to have non-zero pairwise correlations in each phase; here, we have assumed a moderate value of $0.2$, consistent with the specific nature of rare diseases, which do not tend to exhibit high degrees of correlation [28]. In addition, we assume a non-zero pairwise correlation of 0.2 for the market valuation in each phase for the Gaussian copula across the drugs in the portfolio. These modulable assumptions are summarized in Table 7.

**Table 7:** Modulable options of the portfolio Monte Carlo simulation model

Total number of drugs in portfolio	8
Number of drugs starting in preclinical in year 0	8
Number of me-too drugs that are a follow-on of successful drugs in phase 2	0
Number of drugs starting in phase 2 in year 0	0
Correlation of time and cost of development for phases 2 and 3 for the copula within each drug	0.5
Correlation of PoS for the normal copula across drugs for the same phase	0.2
Correlation of commercial value for the normal copula across drugs for the same phase	0.2

### Commercial stage assumptions

To avoid running simulations of different drugs with different rates of prevalence and market entry prices, we do not specify the number of patients nor the price for each drug. Instead, we define the *expected* $\text{rNPV}$ of a single drug after finishing each phase, which we constrain to follow a log-normal distribution. This represents its expected market valuation at the end of each phase. The parameters of this log-normal distribution (Table 8) are based on the values obtained in the Rett syndrome simulation (Table 4). We ensure that the average value and $95\%$ confidence intervals obtained match, and adjust the standard deviation of the distribution, increasing it in successive phases, to account for the fact that the commercial value will have a stronger impact on the expected $\text{rNPV}$ as we approach the end of the drug development process. Of course, this portfolio is specifically based on Unravel Biosciences’ suggested portfolio, and these parameters would need to be modified in the presence of drugs with different developmental aspects.

**Table 8:** Value (rNPV) after each phase (in millions of $)

	Distribution	Average	Standard deviation	Upper bound
Discovery, preclinical development, and phase 1	Log-normal	23.2	21.5	120
Phase 2	Log-normal	117.1	96.3	330
Phase 3	Log-normal	292.3	240.5	690
FDA submission-to-launch	Log-normal	406.5	289.1	880
Overall	Log-normal	8.8	12.8	70

## Appendix B: Key Aspects of a Biotech Startup and Partnership Opportunities

Early-stage biotech companies such as Unravel Biosciences often seek multiple sources of revenue to finance their R&D activities and establish their reputation while remaining competitive. Here, we expand on our discussion of operating models and corporate structures for biotech startups by surveying types of partnerships that are valuable across different development stages, and provide actual examples of biotech–pharma partnership agreements.

### Partnership considerations

Partnerships are integral to the development of the assets of an early-stage company like Unravel, providing financial support and the know-how required to commercialize a drug. The startup would need to find the right balance between leveraging the partners’ expertise in developing drugs and giving up equity to potential partners. We summarize the key partnerships discussed here in Fig. 5.

In its initial stages, the startup should aim to partner with relevant *foundations* to gather disease and patient information, and to raise seed funding. Most foundations are willing to provide financial grants without asking for equity in return, making these partnerships non-dilutive. Additionally, the startup should aim to involve *patient advocacy groups*, which would not only provide important insights that can help to speed and improve scientific advances, but also potentially lead to higher efficacy and faster approvals. The importance of involving patient advocacy groups is even more relevant in the case of rare diseases, where patients and information about the natural history of each disease are scarce.

After the initial discovery phases are complete, the startup should begin to involve biotech-friendly *venture capital firms*, who typically have a higher risk appetite than large pharmaceutical companies and are willing to provide funding in return for some equity in the asset under development. The money would then be used to complete phase 2, and would potentially pave the way for a successful partnership with a large pharma company.

After successful completion of phase 2 and proven efficacy of the asset, the biotech startup’s next partnership would be with a *large pharmaceutical company* that would be willing to collaborate with the startup to further develop the drug. This partnership can have different forms: a general collaboration or partnership, whereby the startup and the pharma company would agree on general terms that would apply to all assets being developed by the startup; an asset-specific out-licensing partnership whereby the startup would defer late-stage development to the large pharma company in return for an upfront out-licensing fee, some milestone payments, and royalties on potential sales in case of FDA approval; and finally an asset sale, whereby the startup would sell the asset completely at the projected valuation.

### Examples of biotech–pharma partnerships

In this section, we survey different examples of biotech–pharma partnerships: in particular, we discuss the partnerships between Gilead and Galapagos [47] and Atomwise and Eli Lilly [48].

#### Gilead–Galapagos partnership

Gilead is an American research-based biopharmaceutical company focused on the discovery, development, and commercialization of innovative medicines, in particular antiviral drugs used in treating HIV, hepatitis B and C, and influenza.

In contrast, Galapagos is a European biotech company known for the discovery and development of small molecule medicines. Its pipeline includes preclinical, phase 1, phase 2, and phase 3 studies, as well as discovery programs in inflammation, fibrosis, and other indications.

*General partnership terms (2019)*. Galapagos will fund and lead all discovery and development autonomously until the end of phase 2. Gilead will then have the option to acquire an expanded license to the compounds developed by Galapagos. If the option is exercised, Gilead and Galapagos will co-develop the compound and share costs equally.

*Financial terms (2019).* Galapagos received a $\$3.95$ billion upfront payment and a $\$1.1$ billion equity investment from Gilead. Gilead will make a $\$150$ million opt-in payment per program and will owe no subsequent milestones. Finally, Galapagos will receive tiered royalties ranging from $20-24\%$ on net sales of all Galapagos products licensed by Gilead.

*Previous partnership for the development of **Filgotinib* *(2015)*. The two companies entered a partnership to develop filgotinib for the treatment of arthritis and other inflammatory diseases [49]. This partnership occurred after phase 2 results showed potential efficacy for patients suffering from rheumatoid arthritis, and Gilead agreed to pay Galapagos an upfront fee of $$\$725$$ million, including a $$\$300$$ million licensing fee and a $$\$425$$ million equity investment in Galapagos. Galapagos was eligible for $$\$1.35$$ billion of milestone payments in later-stage development, as well as tiered royalties starting at $$20\%$$ and a profit split in co-promotion. In 2020, Gilead opted not to continue pursuing regulatory approval in the U.S. and paid Galapagos €160 million (roughly $175 million) to take over most ongoing clinical trials, returning responsibility for the drug in Europe, where it is approved as a treatment for rheumatoid arthritis, as well [50].

#### Atomwise–Eli Lilly partnership

Atomwise is a biotech company that developed an AI technology for quick and effective small molecule drug delivery.^4^ It has partnered with several large pharmaceutical companies and educational institutions, and has raised over $\$50$ million from venture capital firms. In comparison, Eli Lilly & Company is one of the largest pharma companies globally.

*General terms (2019)*. Atomwise will apply its $\text{AI}$-driven technology in support of Eli Lilly’s preclinical drug discovery efforts for up to 10 drugs selected by Eli Lilly. In return, Eli Lilly will provide data to Atomwise on thousands of its molecules and compounds. Finally, Atomwise will have the option to develop compounds from the collaboration that Eli Lilly chooses not to advance into clinical testing itself.

*Financial terms (2019).* Eli Lilly will pay Atomwise up to $\$1$ million in discovery milestones and up to $\$550$ million in total milestone payments tied to achieving later-stage development and commercialization goals.

## Appendix C: Assessment of different corporate structures for biotech startups

In this section, we compare popular options for a corporate structure among early-stage biotech companies using the case of Unravel as an example. Unravel currently possesses a proprietary AI-driven platform that identifies novel drug targets through off-target mechanisms of existing drugs and then develops optimized new molecules. This platform is one of the key assets that differentiates the company from others in the same broad space. The company is also currently developing a drug treatment for Rett syndrome that is expected to enter the clinic in 2023, and has other compounds in the target-to-hit stage. Taking a step into the future, and thinking about the company’s situation with a few additional drugs under development, we explore three different potential corporate structures for the company to consider.

The simplest structure would have all Unravel’s assets (both its drugs and its platform) under one single company. This model does not have any subsidiaries and corresponds to the easiest corporate structure with singular tax filing, bookkeeping, and payroll. The benefit of this simple structure is that it leaves the company with more flexibility to switch to another structure by simply placing assets into subsidiaries, or selling them, if needed. It also provides the company with a high level of discretion to allocate resources among the different assets and allows the company to take advantage of synergies across its different business units and economies of scale when purchasing supplies or utilizing services for each asset. This is particularly meaningful for shared supplies or services such as human resources, legal services, cleaning services and supplies, and office space. Finally, this simple structure can lead to a portfolio financing approach, given that the company will hold all assets under a single entity.

On the end of the other spectrum, Unravel could create a different subsidiary for each drug and for the platform, enabling the company to handle each asset’s special needs separately, and allowing each of the subsidiaries to make independent decisions that are more appropriate for its particular case. The platform would be kept in a different subsidiary, which would allow the company to either monetize it as a single asset via partnering or sell its services to other companies. This structure also allows the company to partner with foundations or associations at a drug-specific level, which may make it easier for those organizations to invest in Unravel, while giving them more transparency and accountability on the use of the resources. This would also allow investors to invest in a single asset without having to worry about the success or failure of the company’s other assets. This structure still allows the company to receive financing at the parent level, although it would not be as straightforward as in the simpler structure described above, given that its subsidiaries might be public or not wholly owned by the parent. There may also be tax treatments that would make investing for the dividends from its subsidiaries more expensive.

Finally, a third option would be for Unravel to keep all its drugs in one subsidiary, and the platform in another. This would allow the company to take advantage of the portfolio financing approach at both the parent and the subsidiary levels. It would also enable the company to take advantage of synergies between the different drugs and allocate developmental resources as it sees fit. Finally, it would also make it easier for the company to compensate the gains and losses from the drugs, since the drug subsidiary entity would have a single tax filing and would not need to worry about having more than $80\%$ ownership of each drug asset. However, this structure might make it harder to attract talent and partner with patient- and disease-centered organizations given that all its resources and drug development efforts would be pooled into a single entity.

The three alternatives discussed here provide an illustration of two extremes of the subsidiary spectrum and an in-between option. However, additional corporate structures exist. It is important to note that each scenario presents advantages and disadvantages, and structures closer to either of these options will tend to have similar benefits and drawbacks. Lastly, subsidiaries can also be based on therapeutic area or target disease. This allows the company to leverage most of the benefits of the extreme cases, while limiting many of the downsides. This is an interesting alternative to the structures described above, provided that the company possesses enough drugs within a therapeutic area or target disease.

## Abbreviations

AI: Artificial intelligence
COGS: Cost of goods sold
FDA: United States Food and Drug Administration
IP: Intellectual Property
NPV: Net present value
PDUFA: Prescription Drug User Fee Act
PoS: Probability of success
QALY: Quality-adjusted life year
R&D: Research and development
rNPV: Risk-adjusted net present value
SG&A: Selling, general, and administrative expenses
U.S.: United States

## Footnotes

1 Website: https://www.unravel.bio/. In the interest of full disclosure, two of the authors—RN and FV—are affiliated with Unravel.

2 Although we focus exclusively on partnerships with large pharmaceutical companies, we should note that small- and medium-sized pharma companies are playing an increasingly larger role in this space. The main points of this analysis should generalize, however specific partnership terms may vary and should be studied on a case by case basis.

3 In the interest of full disclosure, AWL is affiliated with BridgeBio.
